# Primary Seronegative but Molecularly Evident Hepadnaviral Infection Engages Liver and Induces Hepatocarcinoma in the Woodchuck Model of Hepatitis B

**DOI:** 10.1371/journal.ppat.1004332

**Published:** 2014-08-28

**Authors:** Patricia M. Mulrooney-Cousins, Ranjit Chauhan, Norma D. Churchill, Tomasz I. Michalak

**Affiliations:** Molecular Virology and Hepatology Research Group, Division of BioMedical Sciences, Faculty of Medicine, Health Sciences Centre, Memorial University, St. John's, Newfoundland and Labrador, Canada; University of California, San Diego, United States of America

## Abstract

Hepadnavirus at very low doses establishes in woodchucks asymptomatic, serologically undetectable but molecularly evident persistent infection. This primary occult infection (POI) preferentially engages the immune system and initiates virus-specific T cell response in the absence of antiviral antibody induction. The current study aimed to determine whether POI with time may culminate in serologically identifiable infection and hepatitis, and what are, if any, its pathological consequences. Juvenile woodchucks were intravenously injected with inocula containing 10 or 100 virions of woodchuck hepatitis virus (WHV) to induce POI and followed for life or up to 5.5 years thereafter. All 10 animals established molecularly detectable infection with virus DNA in serum (<100–200 copies/mL) and in circulating lymphoid cells, but serum WHV surface antigen and antibodies to WHV core antigen remained undetectable for life. By approximately 2.5–3.5 years post-infection, circulating virus transiently increased to 10^3^ copies/mL and virus replication became detectable in the livers, but serological markers of infection and biochemical or histological evidence of hepatitis remained undetectable. Nonetheless, typical hepatocellular carcinoma (HCC) developed in 2/10 animals. WHV DNA integration into hepatic and lymphatic system genomes was identified in 9/10 animals. Virus recovered from the liver virus-negative or virus-positive phases of POI displayed the wild-type sequence and transmitted infection to healthy woodchucks causing hepatitis and HCC. In summary, for the first time, our data demonstrate that an asymptomatic hepadnaviral persistence initiated by very small amounts of otherwise pathogenic virus, advancing in the absence of traditional serological markers of infection and hepatitis, coincides with virus DNA integration into the host's hepatic and immune system genomes, retains liver pro-oncogenic potency and is capable of transmitting liver pathogenic infection. This emphasizes the role for primary occult hepatitis B virus infection in the development of seemingly cyptogenic HCC in seronegative but virus DNA reactive patients.

## Author Summary

Introduction of highly sensitive molecular assays for detection of hepatitis B virus (HBV) identified the existence of persistent occult HBV infection years after recovery from an episode of hepatitis B and in individuals exposed to HBV but without symptoms and classical markers of infection. Because HBV integrates into human DNA and is a potent human carcinogen, it is postulated that occult HBV infection can be a cause of hepatic cancer in many individuals in which the tumor origin remains currently unknown. A causative relation between occult HBV infection and hepatocarcinoma is highly challenging to investigate in humans since occult HBV persistence is rarely diagnosed with current clinical assays and cancer development takes 15–30 years. However, we have established excellent models of occult HBV infection in the eastern North American woodchucks which are naturally susceptible to a virus closely related to HBV and in which chronic infection advances to liver cancer. In the current study, exploring experimental primary occult infection in woodchucks, we proved that the silently progressing infection, which is not detectable by serological markers, can culminate in hepatocellular carcinoma and that the persisting virus remains infectious, and causes hepatitis and liver cancer when transmitted to virus-naïve hosts.

## Introduction

It is estimated that 370 million people have serologically evident chronic hepatitis B virus (HBV) infection and over 2 billion have been exposed to this virus [1]. Chronic hepatitis B (CHB) frequently (20–25%) advances to cirrhosis and liver failure, while hepatocellular carcinoma (HCC) develops in approximately 5% of the cases [1]. HBV is considered to be primarily hepatotropic; however, it also infects cells of the immune system where it persists for decades even when hepatitis resolves [2]–[4]. These events are closely mimicked in the natural animal model of HBV infection, the eastern North American woodchuck infected with woodchuck hepatitis virus (WHV) [4], [5].

WHV invariably invades the immune system and persists there for life irrespective of whether infection is symptomatic and serologically evident, *i.e.*, serum WHV surface antigen (WHsAg) and antibody to WHV core antigen (anti-WHc) positive, or asymptomatic and serologically silent, *i.e.*, serum WHsAg and anti-WHc nonreactive [6]–[9]. Based on the findings in naturally and experimentally WHV-infected woodchucks, two forms of occult hepadnaviral persistence were uncovered. Secondary occult infection (SOI) continuing after resolution of acute hepatitis (AH) and apparent clearance of serum WHsAg is accompanied by lifelong persistence of anti-WHc and WHV DNA in serum, liver and immune system [5]–[7]. The liver in SOI can display moderate inflammation with periods of normal morphology; nonetheless HCC develops in up to 20% of animals [7]. This form of infection appears to commonly underlie reactivation of hepatitis B in immunocompromised patients and those on cytotoxic therapies [10] and development of HCC in individuals with past exposure to HBV [11], [12].

Another form of hepadnaviral persistence, primary occult infection (POI), was uncovered in offspring of woodchuck dams convalescent from AH and in animals inoculated with WHV doses ≤10^3^ virions [6]–[9]. POI progresses in the absence of identifiable serum WHsAg, anti-WHc and antibodies to WHsAg (anti-WHs), and hepatitis, but WHV and its replication are detectable at low levels in the immune system and sporadically in the liver [6], [9], [13]. Virus-specific T cell, but not B cell, responses are induced and, unlike SOI, protective immunity is not established. Our recent study showed that repeated exposures to liver nonpathogenic WHV amounts, *i.e.*, ≤10^3^ virions, do not culminate in serologically detectable infection and hepatitis or generate immune protection [13]. This type of infection can be suspected in HBV DNA reactive individuals who are seronegative for HBV surface antigen (HBsAg) and antibodies to HBV core antigen (anti-HBc) [14].

The main objective of the current study was to identify lifelong liver pathological consequences of POI, given the known high oncogenic potency of WHV and HBV [4], [15] and the notion that occult HBV infection undetectable by clinical testing might be responsible for HCC of unknown etiology in some cases [11], [12]. We also aimed to identify characteristics of POI regarding virus transmissibility and pathogenic potency in virus-naïve hosts, the status of WHV DNA-host genome integration during POI, and compatibility between virus sequences occurring in the liver virus-negative and the liver virus-positive phases of POI.

## Materials and Methods

### Ethic Statement

All animal experiments and the animal maintenance protocols were performed in compliance with the Institutional Animal Care Committee at Memorial University, St. John's, Newfoundland and Labrador, Canada (protocol identification number 13–159-M) that follows the guidelines and is accredited by the Canadian Council on Animal Care in Science.

### Animals, Induction of POI and Sample Collections

Infection experiments were carried out in 1–2 year old healthy woodchucks housed in the Woodchuck Viral Hepatitis Research Facility at Memorial University, St. John's, Newfoundland and Labrador, Canada. All the animals were captured from a pristine region of Northern Canada. Normal liver function and morphology was ascertained by testing serum biochemical markers of hepatic performance, including sorbitol dehydrogenase (SDH) and γ-glutamyl transferase (GGT), macroscopic inspection of the liver during laparotomy, and by histological examination of liver biopsy taken prior to initiation of the study. Prior exposure to WHV was excluded by negative testing for serum WHsAg and anti-WHc, and by the absence of WHV DNA as determined by highly sensitive polymerase chain reaction/nucleic acid hybridization assays (PCR/NAH) (sensitivity ≤10 copies or virus genome equivalents [vge]/mL or ≤10 vge/µg total DNA) [7]–[9]. WHV/tm3 inoculum (GenBank accession number AY334075 for 3 identical clones) induced serum WHsAg-positive hepatitis in >90% of woodchucks after intravenous (i.v.) administration of doses ≥10^3^ DNase-digestion protected vge, *i.e.*, virions [7]–[9]. WHV/tm5 inoculum (GenBank accession numbers KF874491-3 for 3 clones) was derived from a woodchuck with chronic hepatitis and HCC. WHV/tm5 whole genome sequencing showed 99.7% (3298/3308) and 99.58% (1668/1675) identity in the nucleotide (nt) and amino acid sequences, respectively, when compared to WHV/tm3. Prior to infection, WHV/tm5 was fractionated on cesium chloride gradient to separate virions from free WHsAg, essentially as reported [8]. The recovery of intact virions was ascertained by a DNase-digestion protection assay [2]. To induce POI, 3 animals were i.v. injected with 10 virions of WHV/tm5 and 7 others with 100 virions of WHV/tm3. In addition, 2 animals inoculated with 10^6^ WHV/tm5 virions, 2 inoculated with 10^10^ WHV/tm3 virions, and 2 healthy woodchucks not exposed to WHV, all followed for duration of their lifespan, served as controls. The animals were bled biweekly until 16 weeks post-infection (w.p.i.) and then bimonthly. They were followed for life until senility (animals 1/F, 3/F, 7/M, 10/M and 12/F), HCC development (5/M, 9/M and 11/M), a WHV-unrelated severe health issue requiring termination of follow-up (2/F, 8/M and 14/F) or challenge with 10^10^ virions WHV/tm3 at 66 months post-infection (m.p.i.) (4/F, 6/M and 13/F). The last group of animals was observed for an additional 5.2 months, and bled biweekly until 14 weeks post-challenge (w.p.c.) and then monthly. Liver biopsies were obtained before WHV inoculation and at 6 w.p.i., 8 m.p.i. and then at approximately yearly intervals until autopsy. At autopsy, serum, peripheral blood mononuclear cells (PBMC), liver, bone marrow, spleen, lymph nodes and other organ samples were collected.

### Infection with WHV Derived from Liver Virus-Negative and Liver Virus-Positive Phases of POI

WHV inocula were prepared from the liver virus DNA-negative and the liver virus DNA-positive phases of POI by pooling 24 mL of serum and plasma from 6/M and 7/M, and from 8/M that was liver WHV DNA nonreactive for life. Pellets recovered by ultracentrifugation at 200,000× *g* for 20 hours at 4°C were suspended in 1.3 mL of sterile phosphate buffered saline, pH 7.4. One mL of suspension was i.v. injected into a virus-naïve woodchuck and 0.3 mL used for WHV quantification and sequencing. Thus, A/F animal was injected with 1370 vge from 6/M, C/F with 1460 vge from 7/M, and E/F with 1460 vge from 8/M, all obtained from the liver virus-negative phase of POI. Also, B/F was infected with 2070 vge from 6/M and D/F with 1460 vge from 7/M collected from the liver virus-positive phase of POI. As a control, F/F was injected with 10^10^ virions of WHV/tm3. Plasma and PBMC samples were collected weekly until 8 w.p.i., biweekly until 6 m.p.i, and then bi-monthly. Liver samples were obtained before inoculation, 6–7 w.p.i., 6 m.p.i., then yearly, and at autopsy.

### Sample Processing

PBMC and plasma were harvested from sodium EDTA-treated blood after density gradient centrifugation [7]–[9]. PBMC were cryopreserved, and serum and plasma samples stored at −20°C. Liver samples obtained at biopsy or autopsy were washed, snap frozen and stored at −80°C. For histological examination, liver samples were processed to paraffin, stained and hepatic inflammatory alterations enumerated [7], [8]. Liver neoplastic changes were assessed following morphological criteria reported before [7], [16].

### Serological Assays

WHsAg, anti-WHc were evaluated by enzyme-linked immunosorbent assays (ELISA) reported previously [7]–[9], [13], with sensitivities comparable to or greater than those of clinical assays for detection of equivalent HBV infection markers. The sensitivity of WHsAg ELISA was 3.25 ng/mL while anti-WHc were detectable up to end-point dilution of 1∶64,000. Serum SDH served as a biochemical measure of liver injury and serum GGT as an indicator of HCC development [7], [13].

### WHV DNA, cccDNA and RNA Detections

DNA from 100–400 µL of serum or plasma and from PBMC, liver, bone marrow and lymph nodes was extracted by the proteinase K-phenol-chloroform method [6], [7]. WHV DNA was assessed by direct and, if negative, nested PCR/NAH using primers and conditions reported [7]–[9]. Each sample was tested with 3 primer sets specific for WHV core (C), envelope (S) and X genes [7]–[9]. For nested PCR/NAH detecting WHV covalently closed circular DNA (cccDNA) (sensitivity, ∼10^2^ copies/mL), enzymatic treatment, primers and conditions previously established were applied [8], [13]. Detection of WHV cccDNA was verified by sequencing. WHV RNA was detected by reverse transcription-PCR (RT-PCR; sensitivity, <10 copies/mL) using RNA extracted with Trizol (Invitrogen Life Technologies, Burlington, Canada), treated with DNase (Sigma-Aldrich, Oakville, Canada), and reversely transcribed to cDNA [8], [13]. Each test RNA sample without reverse transcriptase added served as a DNA contamination control [8], [13]. In selected cases, WHV DNA was quantified by real-time PCR (sensitivity, 10–100 vge/mL) using DNA equivalent to 25 µL of plasma or 400 ng of total DNA from cells or tissues, and WHV C and X gene primers. For all assays testing WHV DNA or RNA presence, mock extractions and respective nucleic acid preparations from WHV-positive and WHV-negative woodchuck livers or PBMC were routinely included as controls [6]–[8]. NAH analysis of PCR products was always performed to verify the specificity of virus detection and the validity of controls [6]–[8].

### WHV DNA Sequencing

Low levels of WHV DNA in POI made full virus genome amplifications unfeasible, therefore fragments amplified with C, S and X gene-specific primers and regions spanning WHV polymerase (P) gene between nucleotides (nt) 2948-407 and 1080–1755, X/preC region nt 1503–2122 and preS nt 2948-407 were sequenced (nt positions according to WHV/tm3 AY334075 in GenBank). These regions were selected because they were found to have the most variable sequence based on analysis of full-length WHV genomes using Sequencher v5 (Gene Codes Corporation, Ann Arbor, MI). Amplicons were cloned using the TOPO-TA system (Invitrogen). Ten clones per amplicon were sequenced bidirectionally [17]. The same variants found in at least 2 clones were reported.

### Inverse-PCR for Identification of Virus-Host and Virus-Virus Genome Junctions

Liver and bone marrow DNA of 10–20 kbp purified from agarose served as a template for inverse-PCR (invPCR), as reported [18]. To identify WHV X region-host genome junctions, DNA was digested with *Nsi*-I that cuts WHV at nt 1915 (nt positions according to WHV/tm3 AY334075 in GenBank) and the woodchuck's sequence at unknown sites. To detect WHV preS region-host genome junctions, DNA was treated with *Eco*R-I that cuts WHV/tm3 at nt 3308/1. Diluted digests were circularized with T4 DNA ligase and linearized with *Sph*-I (for X invPCR) or *Pst*-I (for preS invPCR). The possibility of self-ligated virus double-stranded DNA was excluded by *Psi*-I or *Pml*-I digestion. Primers were designed based on consensus sequence of WHV isolates identified in this laboratory (GenBank accession numbers: AY334075, AY6280 and GU734791). For the X region, direct and nested primer pairs were located at nt 1782–1808 and 1718–1737, and 1853–1876 and 1654–1673, respectively. For the preS region, direct primers were located at nt 3231–3253 and 3009–3028, and nested primers at 3202–3222 and 2964–2985. The bands carrying WHV sequences were identified by NAH. DNA was purified by excision from agarose and either directly sequenced bidirectionally or cloned and sequenced. Non-WHV sequences were analyzed with NCBI BLAST and Refseq (National Center for Biotechnology Information, Bethesda, MD). WHV sequences were mapped by aligning with the full-length WHV/tm3 using BioEdit (Ibis Biosciences, Carlsbad, CA).

### Accession Numbers

WHV sequences derived from the liver WHV-negative and liver WHV-positive phases of POI reported in this study were submitted to GenBank under accession numbers KJ755421 for woodchuck 6/M and KJ755420 for 7/M. WHV sequences identified in plasma and spleen of 8/M animal with POI have GenBank accession numbers KJ755405, KJ755406, KJ755410, KJ755411, KJ755415 and KJ755416, while those in E/F woodchuck injected with plasma inoculum derived from 8/M animal have GenBank accession numbers KJ755407-KJ755409, KJ755412-KJ755414, and KJ755417-KJ755419. WHV genome-woodchuck DNA integration sites identified in livers and bone marrows of animals with POI which developed HCC have accession numbers KG817076-85, KG817088, KG817089, KG817091 and KG817092, and those found in livers, PBMC and lymphoid tissues in woodchucks with POI without HCC have accession numbers KG817074, KG817075, KG817086, KG817087, KG817090, and KG817093-99. The sequence of woodchuck HCC H19 gene fragment identified in this study has GenBank accession number KG8117082.

## Results

### Exposure to a Single Small Dose of WHV Establishes Lifelong Serologically Silent but Molecularly Evident Infection

**Figure 1:**
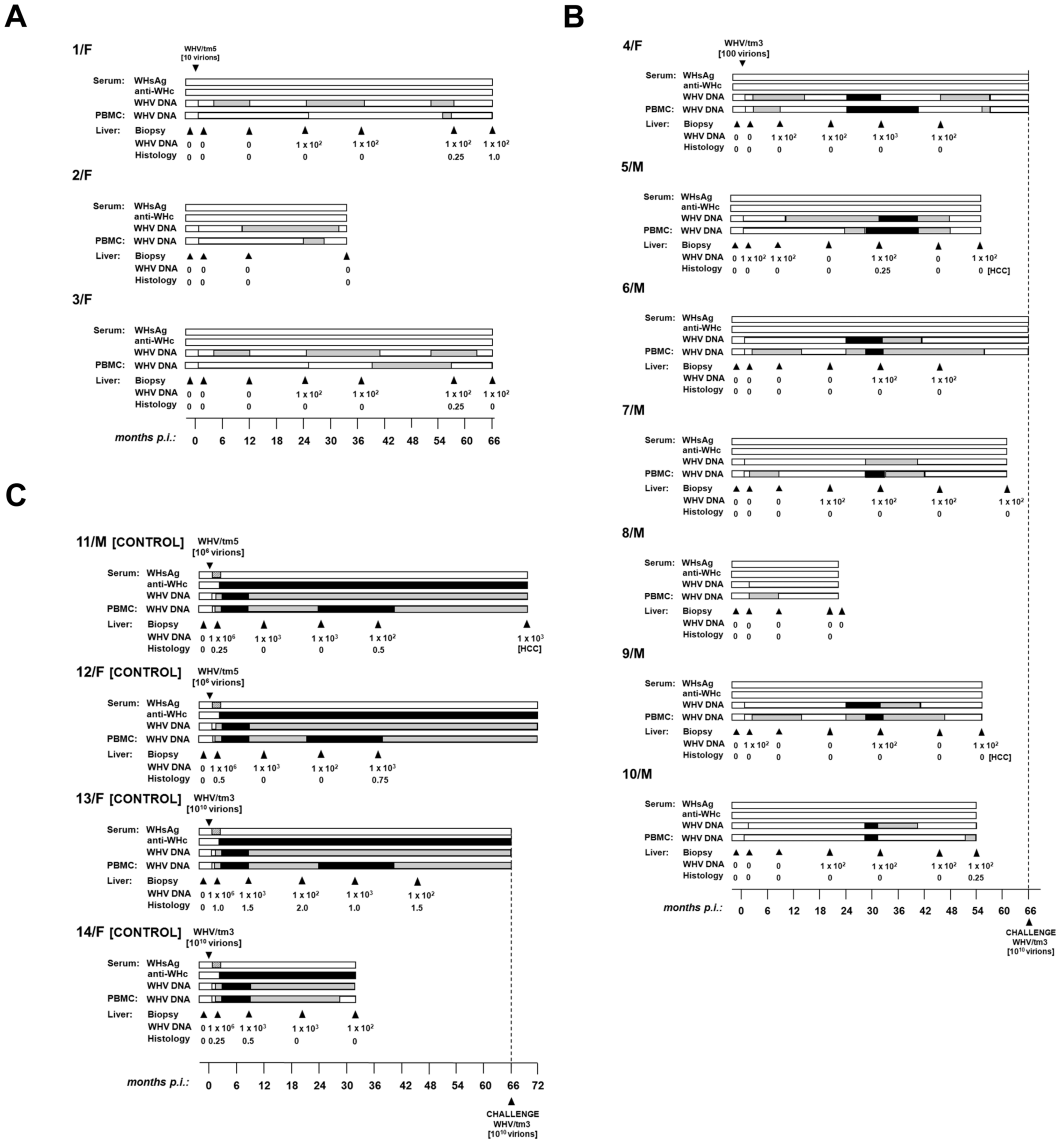
Lifelong profiles of serological markers of WHV infection and WHV DNA detection in serum, PBMC and liver tissue samples, and the results on liver histology in woodchucks injected with a single dose of 10 WHV/tm5 or 100 WHV/tm3 virions and in control animals infected with a liver pathogenic dose of 10^6^ or 10^10^ virions of the same inocula, respectively. (A) Three healthy, WHV-naïve animals i.v. injected with 10 virions recovered after isopynic banding from WHV/tm5 inoculum were followed for life or up to 66 months post-infection (p.i.). (B) Seven healthy, WHV-naïve animals were i.v. injected with 100 DNase-digestion protected virions of WHV/tm3 inoculum. The animals were followed for life or until challenge with a liver pathogenic dose of 10^10^ WHV/tm3 virions at 66 months p.i., as indicated by dotted line. (C) Two healthy woodchucks were injected with 10^6^ WHV/tm5 virions and 2 others with 10^10^ WHV/tm3 virions as controls and followed for life or until challenge with WHV/tm3. The appearance and duration of WHsAg (dotted bars) and anti-WHc (black bars) reactivity in sequential serum samples and WHV DNA in serum and PBMC samples are shown. The estimated levels of WHV DNA detected in sera and PBMC are as follows: white bar, <10 vge per mL or µg DNA, grey bar, 100–200 vge per mL or µg DNA, and black bar, 200–1000 vge per mL or µg DNA. Liver biopsies were obtained at the time points indicated by the solid black arrowheads and their estimated loads of WHV DNA are presented in vge/µg of total DNA. Liver inflammatory alterations were graded by histology on a scale from 0 to 3.

**Figure 2:**
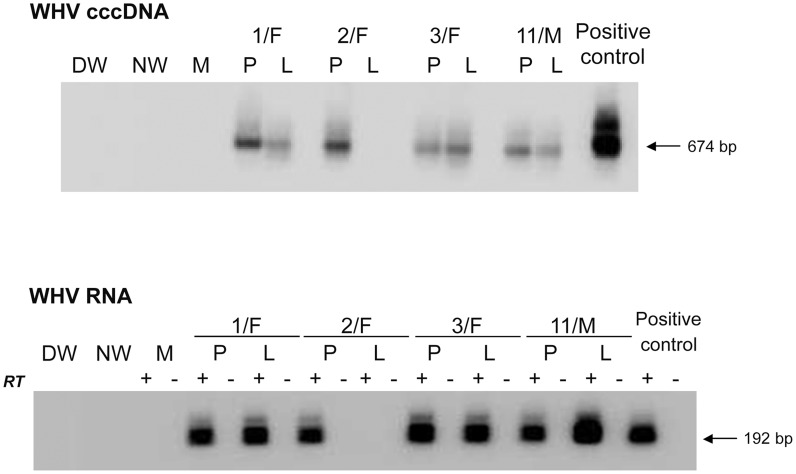
Detection of WHV cccDNA and WHV RNA in liver and PBMC samples obtained at 34–40 months post-infection from woodchucks with POI established by inoculation with 10 WHV/tm5 virions. For detection of WHV cccDNA (top panel), total DNA from PBMC (P) or liver (L) was digested with a single-strand-specific nuclease and amplified by PCR with primers spanning the nick genomic region of WHV. Southern blot hybridization was applied to validate controls and confirm specificity of the 674-bp amplicons. For the detection of WHV RNA (bottom panel), DNase-treated total RNA was reverse transcribed (RT+) or not (RT−) prior to PCR amplification with WHV X gene-specific primers. The specificity of 192-bp amplicons and controls was confirmed by Southern blot hybridization analysis. In both panels, contamination controls consisted of water added to direct (DW) and nested (NW) reactions instead of test DNA or cDNA. Mock samples (M) were extracted and treated as test samples in all steps. DNA and RNA from the liver of a serum WHsAg-positive woodchuck with chronic hepatitis served as positive controls, respectively.

**Figure 3:**
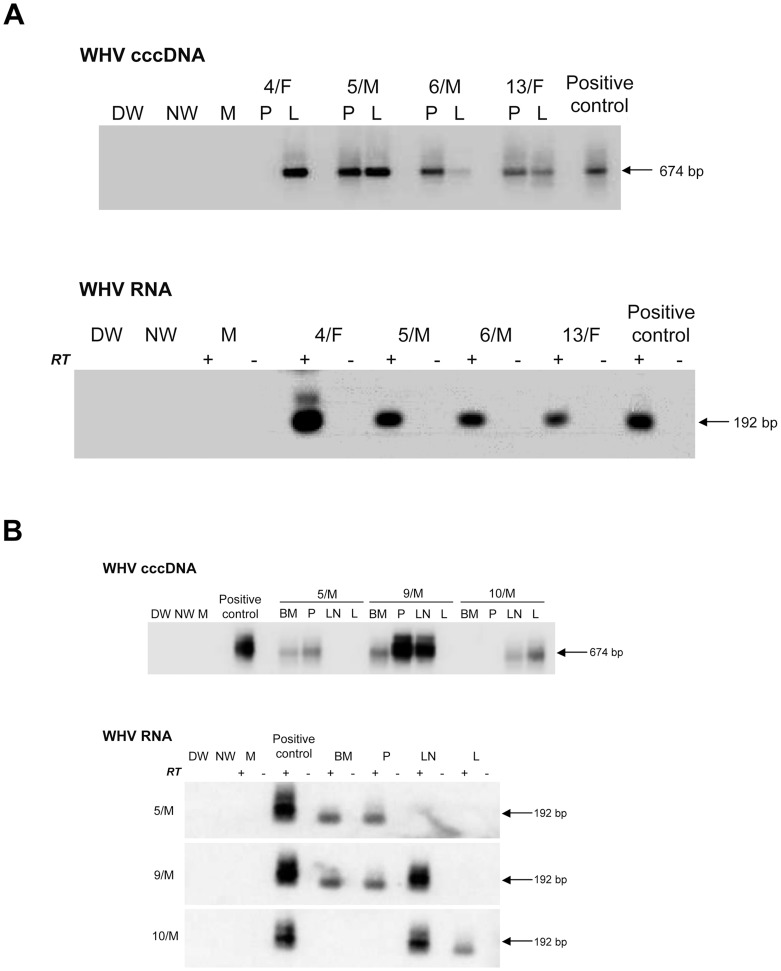
Detection of WHV cccDNA and RNA in liver and lymphoid cell and tissue samples obtained from woodchucks with lifelong POI established after inoculation with 100 virions of WHV/tm3. (A) Identification of WHV cccDNA in PBMC (P) and liver (L) biopsies (top panel) and WHV RNA in the liver biopsies (bottom panel) obtained at 32 months post-infection from 4/F, 5/M and 6/M with POI and from control animal 13/F. (B) Detection of WHV cccDNA (top panel) and WHV RNA (bottom panel) in bone marrow (BM), PBMC (P), lymph node (LN) and in liver (L) samples collected at autopsy from woodchucks 5/M, 9/M and 10/M with POI followed for 54–55 months p.i. with 100 virions of WHV/tm.3. Procedural details, specificity and contamination controls, and their verification were as those described in the legend to Figure 2 and in Materials and Methods.

Animals inoculated with 10 or 100 virions of WHV/tm5 or WHV/tm3, respectively, showed no serological evidence of WHV infection for up to 5.5 years p.i., as revealed by undetectable serum WHsAg and anti-WHc (Figs. 1A and 1B). Nonetheless, WHV DNA was detected in serum/plasma and PBMC throughout the entire follow-up at levels of 100–200 vge/mL or <10^3^ vge/µg cell DNA, respectively (Figs. 1A and 1B). In contrast, woodchucks injected with 10^6^ or 10^10^ virions developed transient serum WHsAg positivity, anti-WHc for life, and biochemical (not shown) and histological evidence of self-limited AH (SLAH) (Fig. 1C). WHV cccDNA and/or WHV RNA were identified in PBMC (Fig. 2 and Fig. 3A) throughout the lifespan and in lymphoid organs at autopsy in woodchucks with POI (Fig. 3B), similarly as in animals with lifelong SOI continuing after SLAH and as reported [7]–[9]. Sequential plasma or serum, liver and PBMC samples from healthy WHV-naïve woodchucks serving as controls remained WHV DNA negative when tested by nested PCR/NAH, while the animals liver and PBMC samples were WHV RNA nonreactive by nested RT-PCR/NAH during the entire observation period (data not shown).

### POI Engages Liver over Time and Causes HCC

**Figure 4:**
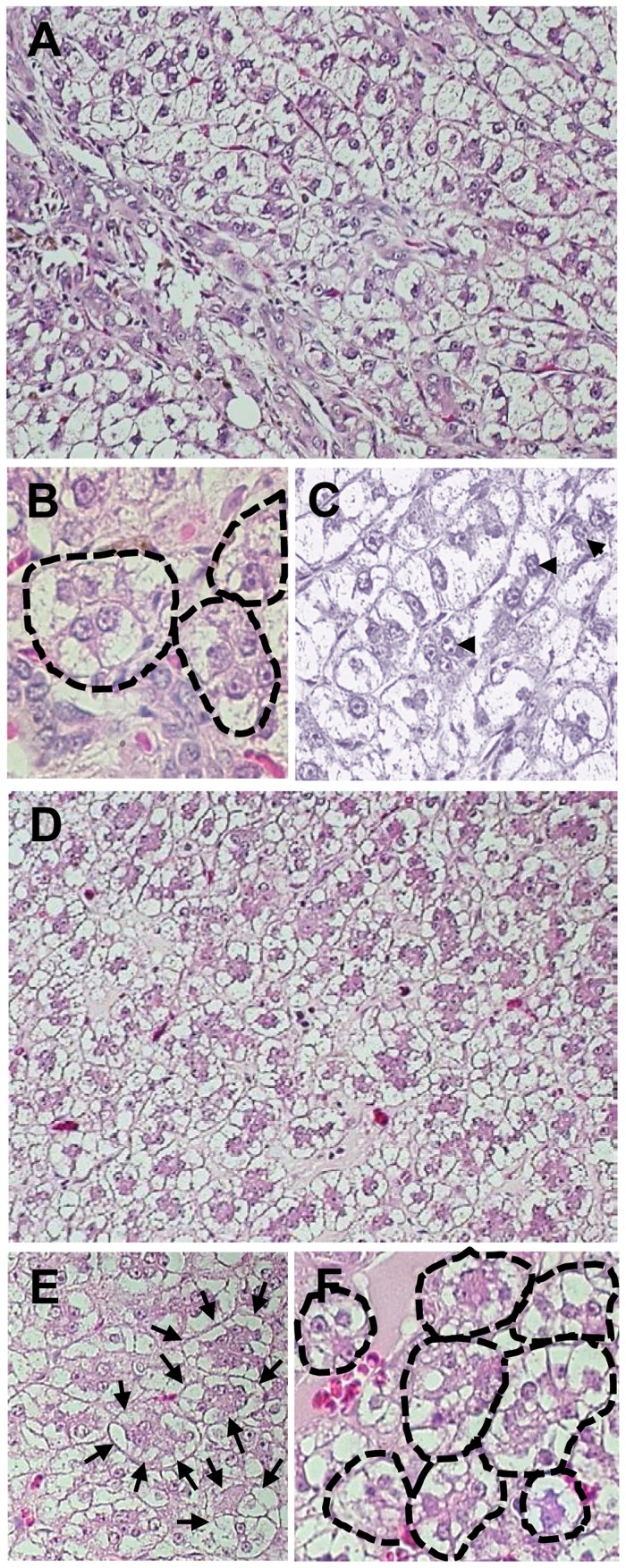
Histological characteristics of HCC in 5/M and 9/M woodchucks at 55 months post-inoculation with 100 WHV virions. (A–C) Representative alterations encountered in cancerous liver tissue of animal 5/M and (D–F) in animal 9/M. Well-defined trabeculae formed by hepatocyte-like cells are evident and demarked with dashed lines (B and F) or by arrows (B). Arrow heads point at frequently observed binuclear cells resulting from cell divisions (C). Accumulation of lipid droplets is apparent in almost all hepatocytes of both animals. Hepatic tissue was obtained at autopsies done in the late fall at the time when storage of fat in the liver is completed prior to physiologically occurring hibernation, although the animals were awake and physically active during the study period. Paraffin sections stained with hematoxylin-eosin. Original magnifications x100 for A and D and x250 for B, C, E and F.

It was not until 32 to 40 m.p.i. that all animals injected with 10 or 100 virions became consistently liver WHV DNA reactive (Fig. 1) and showed evidence of hepatic WHV replication, *i.e.*, detection of WHV cccDNA or WHV RNA or both (Fig. 2). However, 9/M showed transiently a low level of virus DNA in the liver at 6 w.p.i., while 1/F and 5/M were reactive from 26 m.p.i and 6 w.p.i. onwards, respectively (Figs. 1A and 1B). Liver samples from 2/F and 8/M were consistently WHV DNA negative, even up to autopsy performed at 22 and 34 m.p.i., respectively. Liver histology and serum SDH levels remained entirely normal during the whole follow-up, except minimal inflammatory lesions limited to a few portal areas found at 32 m.p.i. in 5/M and at autopsy in 1/F and 10/M (Fig. 1). Despite this infection pattern, typical multinodular HCC has developed in 5/M and 9/M at 55 m.p.i. (Fig. 1B). The diameter of tumor nodules ranged between 2–3 mm (numerous) to 1.5–2 cm (singular) and they were spread throughout the entire livers. Histological examination revealed foci of well-differentiated HCC with hepatocytes arranged in trabeculea (Fig. 4) and, occasionally, with regions of compact cancer tissue. The HCC appearance coincided with moderately elevated serum GGT levels (data not shown). Control woodchucks injected with liver pathogenic doses of WHV showed transiently elevated serum SDH (data not shown) and SLAH followed by SOI accompanied by persistent low-level WHV replication in both liver and PBMC, and intermittent minimal to mild liver inflammation (Fig. 1C), as reported [7], [9]. One of the woodchucks (11/F) inoculated with 10^6^ virions of WHV/tm5 developed HCC at 70 m.p.i. (Fig. 1C). Healthy controls not exposed to WHV had normal serum SDH and GGT levels during follow-up. Their livers remained normal during their lifespan when inspected macroscopically during laparotomies and by histological examination of serial biopsies obtained at approximately yearly intervals (data not shown).

### WHV from Liver Virus-Negative and Liver Virus-Positive Phases of POI Is Infectious, Liver Pathogenic and Displays Essentially the Same Sequence

**Figure 5:**
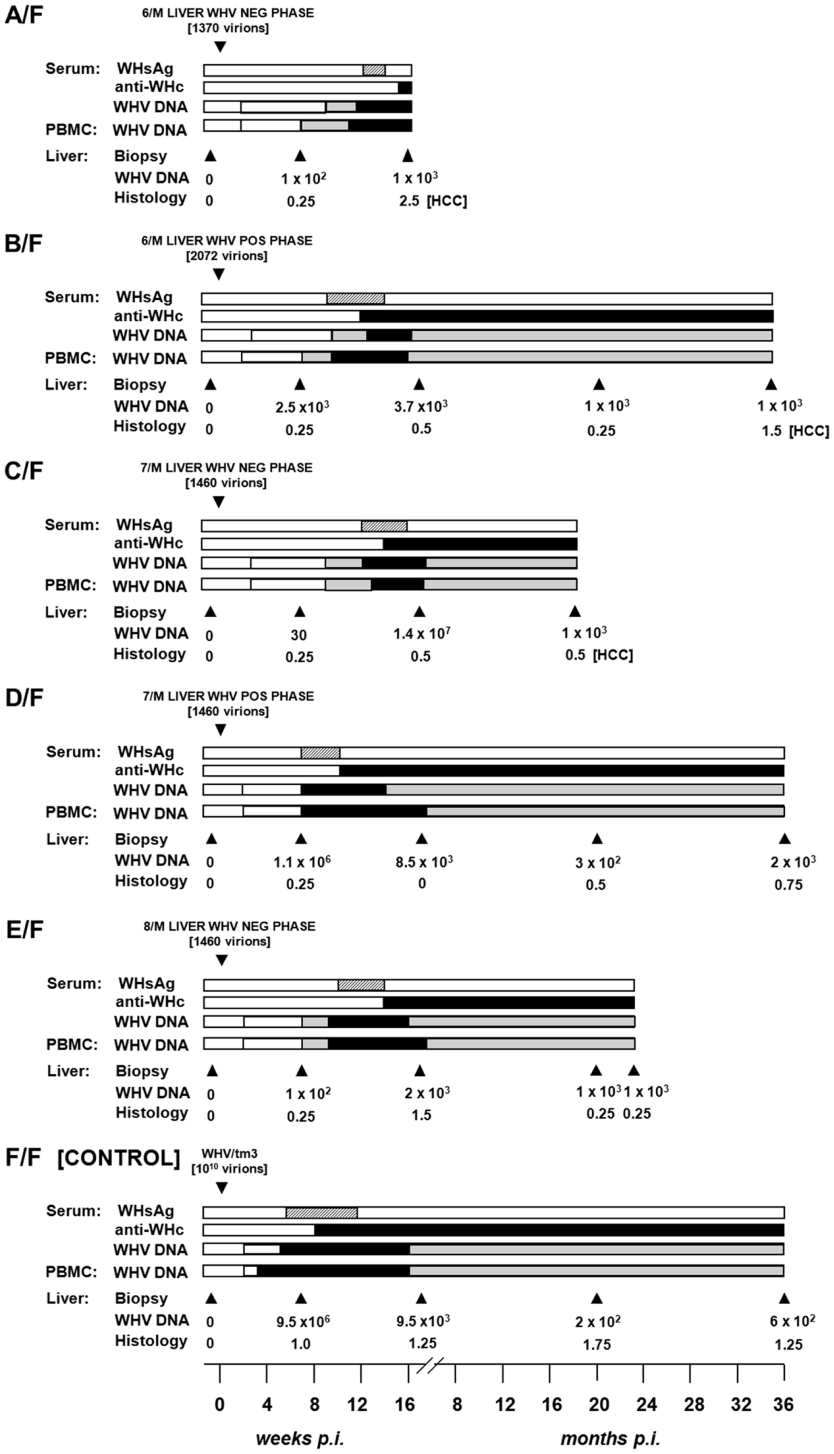
Serological profiles of WHV infection, detection of WHV DNA in sequential serum, PBMC and liver tissue samples, histological degree of hepatitis, and detection of HCC in initially healthy, WHV-naïve woodchucks inoculated with circulating WHV derived from the liver virus-negative and the liver virus-positive phases of POI. Animals were i.v. injected with indicated amounts of WHV, presented in virus genome equivalents (vge), recovered by ultracentrifugation of pools of plasma and serum from the liver WHV-negative and the liver WHV-positive phases of POI from 6/M and 7/M, and from the liver WHV-negative phase of POI from 8/M woodchuck. A control F/F animal was injected with 10^10^ WHV/tm3 virions. The appearance and duration of serum WHsAg and anti-WHc positivity, the estimated WHV DNA loads in sera, PBMC and liver biopsy and autopsy samples, and liver morphological alterations were presented as indicated in the legend to Figure 1.

To determine whether virus persisting as POI retained its infective and pathogenic properties, WHV recovered by ultracentrifugation from pooled serum/plasma collected from the liver WHV-negative or the liver WHV-positive POI phases was administered at doses between 1370 and 2070 virions to virus-naïve woodchucks. All animals developed transiently serum WHsAg-positive infection from 57–84 d.p.i. lasting for up to 113 d.p.i (Fig. 5). The WHsAg appearance was delayed by 22–49 days when compared to F/F control injected with 10^10^ virions. Anti-WHc became detectable at 70–113 d.p.i. (at 57 d.p.i. in F/F) and persisted to the end of follow-up. In animals inoculated with WHV from the liver virus-negative phase of POI, hepatic WHV load at 7 w.p.i. ranged from 30 to 100 vge/µg DNA, whereas in those with WHV from the liver virus-positive phase between 2.5×10^3^ and 1.1×10^6^ vge/µg DNA (9.5×10^6^ vge/µg DNA for F/F). However, subsequent liver biopsies showed comparable WHV DNA levels ranging between 2×10^2^ and 2×10^3^ vge/µg DNA. Similar WHV DNA loads were detected in sera (10-10^2^ vge/mL) and PBMC (10-10^2^ vge/µg DNA) from the beginning of infection regardless of the inoculum source, but these levels subsequently increased by 10–100-fold. All animals (n = 5) developed mild to minimal hepatitis that persisted through the observation period (Fig. 5). Interestingly, 3 of them, including two inoculated with WHV from the liver virus-negative POI phase, developed HCC within 4.5 to 35 m.p.i. accompanied by a variable degree of hepatitis (Fig. 5).

To recognize whether initiation of the liver virus-positive phase of POI might be related to the emergence of a specific WHV variant, 2060-bp of WHV sequences derived from the liver virus-negative and liver virus-positive phases of POI from 6/M and 7/M were compared. The results showed that the WHV sequence from the liver-virus negative phase of 6/M differed only by one non-synonymous mutation in the preC region when compared to that of the virus from the liver virus-positive phase (Table 1). When WHV sequences from the equivalent phases from 7/M were compared, WHV from the liver virus-negative period showed 5 non-synonymous mutations not encountered in the virus from the liver WHV-positive phase (Table 1). However, none of the mutations were compatible with that identified in the preC region of 6/M WHV sequence, suggesting that unlikely a unique hepatotropic variant initiated the liver virus-positive phase of POI.

**Table 1:** Unique non-synonymous mutations identified in circulating WHV derived from the liver virus-negative phase but not from the liver virus-positive phase of POI from 6/M and 7/M woodchucks.

	PreS	Polymerase	X	PreC
	766 bp (nt 2949-407)	1441 bp (nt 2949-407 & 1080–1755)	425 bp (nt 1503–1928)	212 bp (nt 1910–2122)
3/M	n.d.	n.d.	n.d.	V25F
4/M	A91N, T93N, P181L	S279K, N281L	n.d.	n.d.

For each of the WHV genome fragments sequenced, its length (bp) and location (nt) were enumerated according to the WHV/tm3 sequence (GenBank accession number AY334075). The amino acid change and location of each of the non-synonymous mutations identified were based on the predicated amino acid sequence of WHV/tm3. Only variants detected in 2 or more of 10 clones are reported.

Abbreviation: n.d. not detected.

To determine whether WHV derived from the liver-virus negative POI phase retained its sequence after administration to a virus-naïve host, WHV sequences in inoculum and spleen from 8/M, which remained liver virus-negative until autopsy (Fig. 1B), and WHV from plasma, PBMC and liver from E/F, which was injected with 8/M inoculum (Fig. 5), were compared to each other and to WHV/tm3. This analysis showed that 2060-bp of the WHV sequence from 8/M inoculum and spleen displayed very few point mutations when compared to WHV/tm3 (11/2060), 8 conferred amino acid changes and 6 occurrrd in both samples (Table 2). WHV sequences from serum and liver of E/F were highly compatible to that of 8/M inoculum, while E/F PBMC showed a number of non-synonymous variants which were unaccounted for in WHV/tm3 inoculum (n = 31), 8/M inoculum (n = 25) or E/F serum or liver (n = 21) (Table 2), suggesting that the virus after transmission propagated most actively in the lymphoid cells.

**Table 2:** Non-synonymous mutations identified in WHV sequence of the inoculum prepared from the liver WHV-negative phase of POI and in the spleen from 8/M donor and in serum, PBMC and liver of E/F recipient injected with this inoculum.

	PreS	Polymerase	X	PreC
	766 bp (nt 2949-407)	1441 bp (nt 2949-407 & 1080–1755)	425 bp (nt 1503–1928)	212 bp (nt 1910–2122)
***Donor 5/M***				
Inoculum	V179L (0.13%)	S368T, G672D, I689M (0.21%)	T12A, S22G (0.47%)	F11L, G37D (0.94%)
Spleen	V179L (0.13%)	N289Y, S368T, G672D, I689M (0.28%)	T12A, S22G (0.47%)	F11L (0.47%)
***Recipient E/F***				
Serum	P103L, V179L (0.26%)	S368T, G672D, I689M (0.21%)	T12A, S22G (0.47%)	F11L (0.47%)
PBMC	T26P, V47I, T51N, N83S, P103L, H169Q, D174N, V179L, I189T, Q201L (1.3%)	V204M, H206Y, N214T, D227N, S233L, N234K, R239K, H247Y, T267I, N289Y, L293R, K348T, T359P, K362R, D366N, S368T, I381L, G672D, I689M (1.3%)	T12A, S22G (0.47%)	F11L (0.47%)
Liver	D174N (0.13%)	N289Y, K362R, S368T, G672D, I689M (0.35%)	T12A, S22G (0.47%)	F11L, G37D (0.94%)

For each of the WHV genome fragments sequenced, its length (bp) and location (nt) were enumerated according to the WHV/tm3 sequence (GenBank accession number AY334075). The amino acid change and location of each of the non-synonymous mutations identified were based on the predicated amino acid sequence of WHV/tm3. Only variants detected in 2 or more of 10 clones are reported. The number of amino acid changes found in a given WHV genomic region is presented as percentage of the total number of residues in the sequence analyzed.

### POI Is Accompanied by WHV DNA Integration into Host's Liver and Lymphatic System Genomes

Multiple WHV DNA-host genome junctions were identified in animals with POI which developed HCC (5/M and 9/M) or not (1/F, 2/F, 7/M and 10/M) (Table 3). Among virus-host integrants detected in liver biopsy and autopsy samples from 5/M and 9/M, various host sequences were joined predominantly with WHV X gene and less often with the polymerase (P) gene, and preS region sequences (Table 3). None of the virus-host integration sites was identified more than once in the material investigated; however, particular junctions were frequently found in more than one clone (Table 3). Notably, in HCC tissue from 9/M, the 264-bp host sequence flanked by the virus X gene sequence showed 80% homology with mouse H19 cDNA (GenBank accession number AF214115.1). H19 is a tumor suppressor gene and its knockdown may play a role in HCC development [19]. Further to virus-host junctions, multiple virus DNA rearrangements were identified in liver samples from animals with POI-associated HCC, but less frequently in those without cancer (Table 3). Viral-host junctions were also detected in autopsy bone marrow, lymph node and PBMC samples in all 6 animals analyzed (Table 3).

**Table 3:** Virus-host and virus-virus DNA junctions detected in livers and lymphoid tissues and cells of woodchucks with lifelong primary occult WHV infection.

Subgroup/Animal no.	Tissue type	Time of sample acquisition (m.p.i.) detected no.	Overall virus-host DNA junction detected no.	Characteristics of virus-host DNA junction			Overall virus-virus junction
				WHV sequence*	Host sequence length (bp)	Host sequence identity	
***With HCC***							
5/M	Liver autopsy	55	2	S374-380	36	n.d.	2
				S301-306	236	n.d.	
	Bone marrow	55	2	S575-569	272	n.d.	6
				X1590-1583	170	n.d.	
	Liver biopsy	32		PS132-138	263	n.d.	2
	Liver biopsy	20	2	X1877-1916	69	n.d.	0
				P1338-1441	59	n.d.	
9/M	Liver autopsy	55	3	X1782-1808	246	n.d.	6
				X1782-1810	264	H19; mouse 80%	
				P1149-1156	276	n.d.	
	Bone marrow	55	1	X1513-1627	83	n.d.	0
	Liver biopsy	46	2	X1674-1386	82	n.d.	0
				X1632-1317	35	n.d.	
	Liver biopsy	32	1	X1898-2060	66	n.d.	0
***Without HCC***							
1/F	Bone marrow	66	1	P1382-C2335	241	n.d.	4
	PBMC	66	1	X1854-1861	198	n.d.	2
2/F	Lymph node	34	4	C2039-2044	211	n.d.	6
				P1479-1491	146	n.d.	
				S351-343	86	n.d.	
				PS56-61	78	n.d.	
8/M	Liver autopsy	22	1	X1636-P1343	219	n.d.	0
	Bone marrow	22	2	X1636-1501	177	n.d.	0
				X1853-1915	123	n.d.	0
							
10/M	Liver autopsy	72	1	X1627-P1151	39	n.d.	0
	Bone marrow	72	2	X1636-P1088	88	n.d.	0
				X1616-1672	130	n.d.	0

* WHV X, S, P gene or preS (PS) genomic region sequence flanking virus-host junction.

Abbreviations: m.p.i., months post-infection; no., number; n.d., not determined; n.a., not applicable.

### Long-Term POI Does Not Protect from WHV Reinfection and Hepatitis

**Figure 6:**
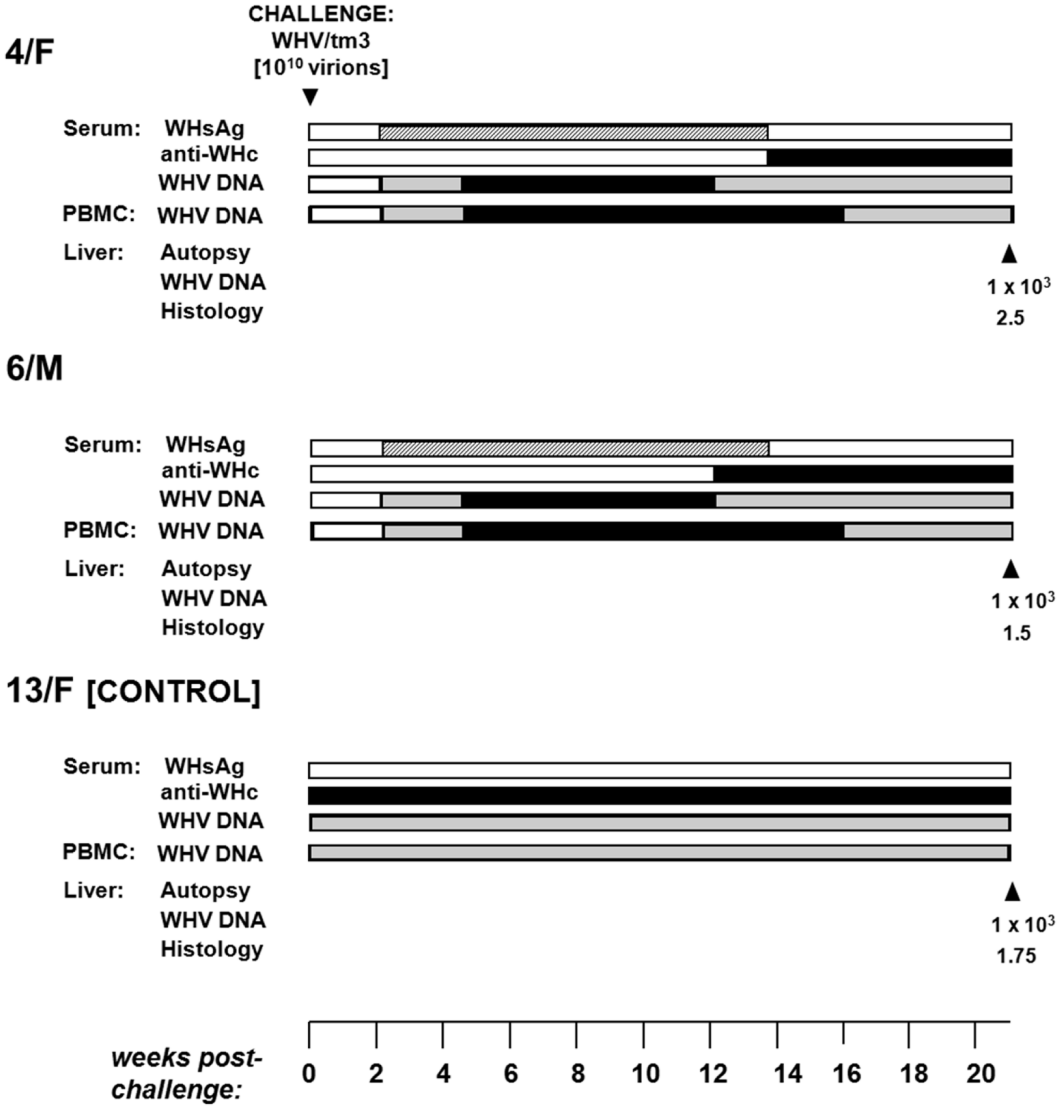
Profiles of serological markers of WHV infection and WHV DNA detection in serum, PBMC and liver tissue samples, and the results on liver histology in 4/F and 6/M woodchucks with POI and in a control 13F animal with SOI after challenge with a single 10^10^ virion dose of WHV/tm3. At 66 months post-injection with 100 or 10^10^ virions of WHV/tm3 (see Figure 1), each woodchuck was challenged with 10^10^ WHV/tm3 virions. The appearance and duration of serum WHsAg and anti-WHc positivity, the estimated WHV DNA loads in sera, PBMC and liver autopsy samples, and liver morphological alterations were presented as described in the legend to Figure 1.

4/F and 6/M with POI lasting for 5.5 years were challenged with 10^10^ virions of WHVtm3 to determine whether the animals might be protected from reinfection. Both woodchucks became serum WHsAg positive at 2 w.p.c. and remained positive until 18 w.p.c. (Fig. 6). Anti-WHc became detectable from 13–14 w.p.c. Serum SDH levels increased and peaked at 8–14 w.p.c., while liver histology displayed moderate to severe AH (Fig. 6). WHV DNA levels in serum and PBMC were similar to those detected in control animals over the course of SLAH (Fig. 1C). Additionally, samples collected from 4/F and 6/M at autopsy, when serum WHsAg was undetectable, displayed low levels of WHV DNA in serum, liver and lymphatic organs, implying existence of SOI. Thus, both 4/F and 6/M developed acute hepatitis despite being persistently infected with WHV at a low-level. This was in contrast to 13/F with established SOI, which was protected from challenge (Fig. 6), similarly as previously reported [7], [9], [13].

## Discussion

We uncovered that minute amounts of hepadnavirus establish infection that persists indefinitely in the woodchuck model of hepatitis B in the absence of conventional serological markers of infection and hepatitis, but is detectable molecularly when sensitive virus nucleic acid-specific amplification assays are applied. We also documented that this form of asymptomatically hepadnaviral carriage, designated previously as POI [5], [9], [13], has both pathogenic and epidemiological relevance since it can lead to the development of HCC and, under certain conditions, transmit infection and cause hepatitis and HCC in virus-naïve hosts. Another important finding, albeit expected, was that POI is associated with hepadnavirus DNA integration into the hepatic and immune system DNA, which likely underpins liver oncogenic potency of the virus persisting during the course of this asymptomatic form of hepadnaviral carriage.

The results from the current investigations also showed that during POI, WHV replication expands to the liver with time, but the level and/or type of cells infected appear to be inadequate to trigger hepatitis. In previous studies, woodchucks with experimental POI were followed for up to 25 m.p.i. without detection of WHV in the liver or evidence of HCC, while WHV replication was detectable in circulating and organ lymphoid cells [9], [13]. Also, offspring born to woodchuck dams with SOI, which acquired lymphatic system-restricted POI, did not show liver engagement and the development of HCC during the 42-month observation period [6]. Although there might be several factors contributing to the development of HCC during POI, the virus spreading to the liver and the extended period of POI follow-up appear to be critical.

WHV genome fragments from the liver virus-negative and the liver virus-positive POI phases showed essentially the same predicted amino acid sequences (Table 1), which also were highly compatible to that of wild-type WHV inocula used to induce POI in this study. We analyzed more than 62% of the total WHV sequence, including virus regions identified as having the highest sequence variability based on our preceding analysis of the complete WHV sequences reported in GenBank. We used this approach because the trace quantities of WHV found during POI and the inherently lower sensitivity of the extended PCR amplifying long WHV sequences made full virus genome amplification not feasible. We also identified that the WHV/tm3 sequence, as far as we were able to determine, was conserved in the animals injected with WHV prepared from the liver WHV-negative or the liver WHV-positive phases of POI, which developed WHV infection engaging both the liver and the lymphatic system. These findings imply that the dual tropism of WHV towards hepatocytes and immune cells is unlikely due to the existence of cell type-specific viral variants but is an intrinsic propensity of the naturally occurring virus. This is consistent with data from *in vitro* infection experiments in which the same wild-type WHV was serially passaged in cultured woodchuck hepatocytes and lymphoid cells [17]. This issue has not yet been investigated in HBV infection.

Our previous studies showed that serologically overt WHV infection coinciding with hepatitis is resultant from *i.v.* administration of WHV doses greater than 1×10^3^ virions (liver pathogenic doses), while lower doses of the same wild-type virus (liver non-pathogenic doses) consistently induced POI in woodchucks [9], [13]. In the current study, concentration by ultracentrifugation of virus from animals with POI to levels above the previously identified liver pathogenic threshold was accomplished and, as documented, the recovered virus readily induced serologically overt infection and hepatitis upon transmission to virus-naïve animals (see Fig. 5). It appears that the inability of WHV to engage the liver during the initial phase of POI was related to the very low quantities of the produced virus which, however, can be temporally augmented to the level sufficient to invade the liver. In this regard, we detected a transient increase in plasma WHV load to approximately or above 1×10^3^ vge/mL that preceded detection of WHV DNA and its replication intermediates in hepatic tissue. It can be assumed that this temporal increase in circulating WHV was adequate to engage the liver during later phase of POI, which prior to that was restricted to the lymphatic system. This appears to be consistent with identification of a 100 to 1000-fold greater affinity of synthetic analogues of WHV cell binding site for activated woodchuck lymphoid cells than woodchuck hepatocytes, suggesting that very low quantities of virus may preferentially invade the immune system [20], [21].

The mechanism of liver carcinogenesis in hepadnaviral infection is not well understood, but it is likely a multistep process in which persistent virus infection and virus genome integration into host DNA are among the principal contributors [22]. Random HBV DNA integration into the liver genome was found in up to 22% of patients with CHB and is a typical finding in HBV-related HCC (>80% patients) [22]–[24]. On the other hand, the status of HBV DNA integration into HCC DNA coinciding with occult HBV infection was only occasionally investigated and mainly in cirrhotic patients [11], [12]. Nonetheless, the data convincingly showed that HBV DNA integrates into both HCC and non-HCC liver DNA in serum HBsAg-negative patients, with or without detectable anti-HBc [11], [12]. In WHV-related HCC, virus DNA insertions were identified in tumors developing during chronic hepatitis and SOI continuing after SLAH [25]. WHV DNA integration was frequently found near the *myc* pro-oncogenes in HCC coinciding with chronic WHV hepatitis [26], [27]. We did not find this relation in woodchucks developing HCC during POI. However, this might become more apparent when a greater number of relevant cases are analyzed. About two-thirds of the virus-host genome junctions detected in this study encompassed the WHV X gene sequence (Table 3). This resembles the predisposition of HBV X gene to integrate into the host genome reported in serum HBsAg-negative patients with HCC [28]. In our study, HCC had developed in the absence of hepatitis and cirrhosis. In contrast to CHB, chronic WHV hepatitis never leads to cirrhosis and very rarely to fibrosis (<1%) [4]. However, the occurrence of HBV-related HCC in the absence of cirrhosis has been reported [28]. The present finding of the POI-associated HCC mimics the human disease situation where HBV-related HCC develops in the absence of apparent chronic liver disease and serological evidence of HBV infection. Notably, the finding of WHV DNA sequence insertions within bone marrow DNA in POI parallels HBV DNA and WHV DNA integration into lymphoid cells and lymphatic organ genomes in CHB and in woodchucks with chronic hepatitis and SOI [29], [30].

This study also revealed that POI during lifelong follow-up did not culminate in serologically apparent infection or hepatitis, and did not induce protective immunity. These findings add new dimensions to the previous investigations on POI [6], [9], [13]. Among others, our previous study showed that repeated *i.v.* injections (12 in total) with 100 WHV virions did not initiate serologically detectable infection or hepatitis, but molecularly evident POI was established and continued until challenge with a liver pathogenic dose (>10^3^ virions) of the same virus inoculum [13]. In the current study (data not shown) and in the previous investigations [9], [13], [31], [32], WHV-specific T cell reactivity occurring in the absence of virus-specific antibody response did not protect from challenge with liver pathogenic doses of WHV (>10^3^ virions). This is in marked contrast to WHV-specific T cell responses coinciding with virus-specific antibodies in SOI continuing after recovery from symptomatic WHV infection and hepatitis which yield total protection against challenge with even massive doses of WHV (>10^10^ virions) [7], [31]. The former may parallel a situation in unvaccinated individuals having repeated contacts with infected persons and intravenous drug users repetitively exposed to small amounts of HBV. Our data implies that these individuals would unlikely become serum HBsAg and anti-HBc reactive or immune to HBV infection, but the development of HCC in such persons cannot be excluded. Nonetheless, the current findings are in contrast to data indicating that one virion of HBV derived from a HBV transgenic mouse was able to induce serologically evident chronic hepatitis in chimpanzee [33]. Differences in the liver pathogenic potency between a single HBV isolate from a transgenic mice and intact, naturally occurring WHV might explain this discrepancy.

Although the existence of POI in humans has not yet been thoroughly investigated, the prevalence of HBV DNA-reactive infection seronegative for HBsAg and anti-HBc has been reported between 0.07 and 7.6% of subjects in different areas of HBV endemicity [12], [34]. It can be expected that HBV POI is much more frequent because the assays available for HBV DNA detection are approximately 10–100-fold less sensitive than these utilized in this study. Further, HBV-specific T cell responses in the absence of serum HBsAg and anti-HBc have been identified in HBV DNA-reactive patients, further supporting that this silent form of HBV infection naturally occurs [14]. It is of note that WHV-specific T cell responses were also examined in the current study and they persisted at borderline levels after a period of heightened reactivity lasting between 6 and 20 w.p.i. (data not shown).

In conclusion, this study revealed the oncogenic capacity and potential epidemiological significance of asymptomatic hepadnaviral carriage initiated by very small amounts of otherwise pathogenic virus that advances in the absence of traditional serological markers of infection and hepatitis. The data emphasize the role for primary occult HBV infection in the development of seemingly cyptogenic HCC in HBV seronegative patients.
